# Metabolic syndrome and the risk of urothelial carcinoma of the bladder: a case-control study

**DOI:** 10.1186/s12885-015-1769-9

**Published:** 2015-10-16

**Authors:** Maurizio Montella, Matteo Di Maso, Anna Crispo, Maria Grimaldi, Cristina Bosetti, Federica Turati, Aldo Giudice, Massimo Libra, Diego Serraino, Carlo La Vecchia, Rosa Tambaro, Ernesta Cavalcanti, Gennaro Ciliberto, Jerry Polesel

**Affiliations:** 1 Unit of Epidemiology, Istituto Tumori “Fondazione Pascale IRCCS”, Via Mariano Semmola, 1 – 80131 Naples, Italy; 2 Unit of Epidemiology and Biostatistics, CRO Aviano National Cancer Institute, via F. Gallini, 2 – 33081 Aviano, Italy; 3 Department of Epidemiology, IRCCS - Istituto di Ricerche Farmacologiche “Mario Negri”, via La Masa, 19 - 20156 Milan, Italy; 4 Department of Medical Statistics, Biometry and Bioinformatics, Fondazione IRCCS Istituto Nazionale Tumori, via A. Vanzetti, 5 – 20133 Milan, Italy; 5 Laboratory of Translational Oncology & Functional Genomics, Department of Biomedical and Biotechnological Sciences, University of Catania, via Androne, 83 – 95124 Catania, Italy; 6 Department of Clinical Sciences and Community Health, Università degli Studi di Milano, via A. Vanzetti, 5 - 20133 Milan, Italy; 7 Department of Urology and Gynecology, Istituto Tumori “Fondazione Pascale IRCCS”, Via Mariano Semmola, 1 – 80131 Naples, Italy; 8 Division of Medicine Laboratory and Clinical Pathology, Istituto Tumori “Fondazione Pascale IRCCS”, Via Mariano Semmola, 1 – 80131 Naples, Italy; 9 Istituto Tumori “Fondazione Pascale IRCCS”, Cappella dei Cangiani, 1 - 80131 Naples, Italy

**Keywords:** Bladder cancer, Diabetes, Metabolic syndrome, Obesity

## Abstract

**Background:**

The Metabolic syndrome (MetS) is an emerging condition worldwide, consistently associated with an increased risk of several cancers. Some information exists on urothelial carcinoma of the bladder (UCB) and MetS. This study aims at further evaluating the association between the MetS and UCB.

**Methods:**

Between 2003 and 2014 in Italy, we conducted a hospital-based case-control study, enrolling 690 incident UCB patients and 665 cancer-free matched patients. The MetS was defined as the presence of at least three of the four selected indicators: abdominal obesity, hypercholesterolemia, hypertension, and diabetes. Odds ratios (ORs) and corresponding 95 % confidence intervals (CIs) for MetS and its components were estimated through multiple logistic regression models, adjusting for potential confounders.

**Results:**

Patients with MetS were at a 2-fold higher risk of UCB (95 % CI:1.38–3.19), compared to those without the MetS. In particular, ORs for bladder cancer were 2.20 (95 % CI:1.42–3.38) for diabetes, 0.88 (95 % CI: 0.66-1.17) for hypertension, 1.16 (95 % CI: 0.80-1.67) for hypercholesterolemia, and 1.63 (95 % CI:1.22–2.19) for abdominal obesity. No heterogeneity in risks emerged across strata of sex, age, education, geographical area, and smoking habits. Overall, 8.1 % (95 % CI: 3.9-12.4 %) of UCB cases were attributable to the MetS.

**Conclusions:**

This study supports a positive association between the MetS and bladder cancer risk.

## Background

Bladder cancer ranks among the 10 highest incident cancers worldwide; it is one of the most frequent malignant tumours of the urinary system, with approximately 420,000 new cases each year among men and women, and a leading cause of cancer-related deaths [1, 2]*.* Incidence rates are three-to-four-fold higher in men than in women, and more than 90 % of cases are urothelial carcinoma of the bladder (UCB). In Italy, standardized incidence rates for bladder cancer are 29.9 and 6.2/100,000 among men and women, respectively [3].

Tobacco smoking is a major risk factor for UCB, being responsible for 30 % to 50 % of cases in both sexes [4]. Other risk factors have been involved in UCB onset, including obesity, hypertension and diabetes [5–7]. The strong association with these medical conditions suggests a possible role of the metabolic syndrome (MetS) in UCB etiology [6, 7]. The MetS is a complex disorder described as a cluster of at least three risk factors for cardiovascular disease, including abdominal obesity, glucose intolerance, high blood pressure, high triglyceride levels and low high-density lipoprotein cholesterol levels [8].

The MetS has been consistently associated to increased risk for several cancers [6], of magnitude ranging from 1.1 to 1.6. Among women, the strongest associations were reported for endometrial, breast (postmenopausal), pancreatic and colorectal cancers [6, 7, 9, 10]. Among men, the strongest associations were with liver cancer, which persisted after adjustment for chronic infection with HBV/HCV [11], renal and colorectal cancer [6, 7, 11, 12].

Although the prevalence of the MetS is increasing worldwide and high rates of UCB are documented in most countries, few epidemiological studies have been published on the relationship between the MetS and bladder cancer in the Mediterranean region. Two recent systematic reviews on the relationship between the MetS and UCB risk reported a positive association in men only [6, 7]. Therefore, to provide further information on the issue in a Mediterranean area, we examined data from an Italian case-control study investigating potential risk factors for UCB.

## Methods

Between 2003 to 2014, we conducted a case-control study on urothelial carcinoma of the bladder within an established Italian network of collaborating centres, including Aviano and Milan in northern Italy, and Naples and Catania in southern Italy [13]. Cases were 690 patients aged 25 years or older (median age: 67 year; range: 25-84 years) with incident UCB admitted to major general hospitals in the study areas. Nearly all UCB (n = 642, 93.0 %) were confirmed by histological testing on tumour tissue specimen from biopsy or surgery. However, cases whose papillary features could not be determined (n = 138, 20 %) were excluded from the analysis of histological subtypes but were included in all other analyses. Overall, 268 UCB (38.8 %) were non-invasive (i.e., TNM pTis/Ta) and 307 (44.5 %) were well or modestly differentiated.

The control group included 690 patients frequency-matched to cases according to study centre, sex, and 5-year age group. Twenty-five controls were excluded after enrolment because of inappropriate admission diagnosis, thus leaving 665 eligible controls (median age: 66 years; range: 27-84 years). Controls were admitted to the same network of hospitals as cases for a wide spectrum of acute, non-neoplastic conditions unrelated to tobacco and alcohol consumption, to known risk factor for UCB, or to conditions associated to long-term diet modification. Overall, 28.9 % of controls were admitted for traumatic disorders, 22.1 % for non-traumatic orthopaedic disorders, 39.3 % for acute surgical conditions, and 9.8 % for other various illnesses. All study subjects signed an informed consent. Study protocol was approved by the Ethic Board of each study hospital (S. Maria degli Angeli hospital, register trial number 8/2004; and CRO Aviano National Cancer Institute, protocol number 590/D).

Trained interviewers administered a structured questionnaire to cases and controls during their hospital stay, thus keeping refusal below 5 % for both cases and controls. The structured questionnaire collected information on socio-demographic factors; lifetime smoking and alcohol drinking habits; dietary habits related to the two years preceding diagnosis/interview; problem-oriented medical history; and family history of cancer. Two specific sections investigated lifetime occupational exposure, and exposure to chemicals known (or suspected) to be related to UCB, including the use of hair dyes [13].

Information on clinical diagnosis of diabetes, drug-treated hypertension, and drug-treated hyperlipidaemia was self-reported and included age at diagnosis [14]. Diseases whose onset was less than one year before the interview were not considered. Likewise, self-reported height and weight one year prior to diagnosis/interview and at 30 and 50 years of age were collected.

Body mass index (BMI) was computed through the Quetelet’s formula (weight divided by squared height – kg/m^2^). The interviewers measured the waist circumference (2 cm above the umbilicus). The presence of abdominal obesity was defined using the International Diabetes Federation (IDF) cut-points (waist circumference ≥ 94 cm for men and ≥ 80 cm for women). Information on waist circumference could not be obtained for technical reason in 157 cases and 192 controls, thus leaving 533 cases and 473 controls for the present analysis shown in Tables 2 and 3. Sensitivity analyses were further conducted on all cases and controls using BMI ≥ 30 kg/m^2^ as a proxy of abdominal obesity in patients missing waist circumference. MetS was determined according to the 2009 joint interim statement [15], as the presence of at least three of the following components: abdominal obesity, diabetes, drug-treated hypertension (as a proxy of elevated blood pressure), and drug-treated hyperlipidaemia (as a proxy of high triglyceride levels).

Odds ratios (ORs) and the corresponding 95 % confidence intervals (CIs) were calculated by means of unconditional logistic regression models, including terms for study centre, sex, 5-year age groups, years of education (i.e., <7, 7-11, ≥12) as a proxy of social status. To adjust for potential confounders (i.e. factors associated to both outcome and exposure), smoking habits (never; former; current: <20; 20+ cigarettes/day) were further included in the model. The test for trend was based on the likelihood-ratio test between the models with and without the linear term, reporting the median values in each strata of the variable of interest. Percent attributable risks (PAR) were computed using the distribution of risk factors among UCB cases [16].

## Results

Most UCB cases were men and aged ≥65 years (Table 1). Cases and controls reported similar education, whereas current tobacco smoking was more frequent among UCB cases than controls (39.8 % and 21.7 %, respectively). Compared to never smokers, subjects smoking ≥20 cigarettes/day showed a seven-fold increased in UCB risk (95 % CI: 4.94-11.41), with a significant risk trend for number of cigarettes (*P < 0.01*).

**Table 1:** Distribution of cases of urothelial carcinoma of the bladder (UCB) and controls, odds ratio (OR) and corresponding 95 % confidence interval (CI) according to socio-demographic characteristics and tumor variables. Italy, 2003-2014

Variables	UCB Cases		Controls		OR (95 % CI)^a^
	n	(%)	n	(%)	
Sex					
Men	595	(86.2)	561	(84.4)	
Women	95	(13.8)	104	(15.6)	
Age (years)					
<55	83	(12.0)	105	(15.8)	
55-59	65	(9.4)	73	(11.0)	
60-64	107	(15.5)	119	(17.9)	
65-69	164	(23.8)	147	(22.1)	
70-74	155	(22.5)	124	(18.7)	
≥75	116	(16.8)	97	(14.6)	
Study centre					
Aviano	242	(35.1)	250	(37.6)	
Milan	241	(34.9)	238	(35.8)	
Naples	129	(18.7)	100	(15.0)	
Catania	78	(11.3)	77	(11.6)	
Education (years)^b^					
<7	292	(42.3)	273	(41.1)	Ref
7-11	224	(32.5)	215	(32.3)	1.10 (0.85-1.44)
≥12	173	(25.1)	177	(26.6)	1.02 (0.76-1.36)
*χ*^2^ for trend; p-value					0.03; *P = 0.86*
Smoking habit^b^					
Never	96	(13.9)	237	(35.6)	Ref
Former	310	(44.9)	284	(42.7)	2.80 (2.06-3.80)
Current					
<20 cig./day	143	(20.7)	87	(13.1)	4.81 (3.32-6.98)
≥20 cig./day	132	(19.1)	57	(8.6)	7.51 (4.94-11.41)
*χ*^2^ for trend; p-value					103.28; *P < 0.01*
Histological subtype					
Non-papillary	103	(14.9)			
Papillary	449	(65.1)			
Not determinable	138	(20.0)			

^a^Adjusted for sex, age (<55; 55-59; 60-64; 65-69; 70-74; ≥75 years), and study centre; ^b^The sum does not add up to the total because of missing values

Compared with people without any MetS components, the ORs were 2.00 (95 % CI: 1.17-3.41) for those with three components and 7.93 (95 % CI: 1.71-36.79) for those with four components (P for trend < 0.01). After adjustment for the other MetS components, patients with diabetes (16.9 % cases and 8.0 % controls) showed a two-fold increase in UCB risk (95 % CI: 1.42-3.38 - Table 2). Likewise, patients reporting abdominal obesity showed a significantly higher UCB risk (OR = 1.63; 95 % CI: 1.22-2.19). No significant association emerged for treated hypertension (OR = 0.88; 95 % CI: 0.66-1.17) and treated hyperlipidaemia (OR = 1.16; 95 % CI: 0.80-1.67).

**Table 2:** Distribution of cases of urothelial carcinoma of the bladder (UCB) and controls, odds ratio (OR) and corresponding 95 % confidence interval (CI), according to indicators of metabolic syndrome (MetS). Italy, 2003-2014

Components	UCB Cases		Controls		OR (95 % CI)^a^	OR (95 % CI)^b^
	n	(%)	n	(%)		
Diabetes mellitus						
No	443	(83.1)	435	(92.0)	Ref	Ref
Yes	90	(16.9)	38	(8.0)	2.22 (1.45-3.39)	2.20 (1.42-3.38)
Drug-treated hypertension						
No	316	(59.3)	279	(59.0)	Ref	Ref
Yes	217	(40.7)	194	(41.0)	0.99 (0.75-1.31)	0.88 (0.66-1.17)
Drug-treated hyperlipidaemia						
No	442	(82.9)	399	(84.4)	Ref	Ref
Yes	91	(17.1)	74	(15.6)	1.21 (0.84-1.73)	1.16 (0.80-1.67)
Abdominal obesity^c^						
No	145	(27.2)	167	(35.3)	Ref	Ref
Yes	388	(72.8)	306	(64.7)	1.65 (1.23-2.21)	1.63 (1.22-2.19)
Nr. of MetS components						
None	83	(15.6)	85	(18.0)	Ref	
1	213	(40.0)	208	(44.0)	1.03 (0.70-1.51)	
2	154	(28.9)	138	(29.2)	1.23 (0.81-1.85)	
3	67	(12.6)	40	(8.5)	2.00 (1.17-3.41)	
4	16	(3.0)	2	(0.4)	7.93 (1.71-36.79)	
*χ*^2^ for trend; p-value					11.45; *P < 0.01*	
Increment of 1 MetS component					1.29 (1.11-1.49)	
Indicators of MetS^d^						
No	450	(84.4)	431	(91.1)	Ref	
Yes	83	(15.6)	42	(8.9)	2.09 (1.38-3.19)	
"Without diabetes"	29	(5.4)	26	(5.5)	1.16 (0.65-2.07)	
"With diabetes"	54	(10.1)	16	(3.4)	3.63 (1.99-6.61)	

^a^Adjusted for sex, age (<55; 55-59; 60-64; 65-69; 70-74; ≥75 years), study centre, education (<7; 7-11; ≥12 years), and tobacco smoking (never; former; current: <20; 20+ cigarettes/day); ^b^Separate components were additionally adjusted for the other MetS components; ^c^According to IDF cut-points for waist circumference. ^d^Defined as the presence of at least three out of four MetS components

Compared to patients without indication of MetS (i.e., with two or less MetS indicators), those with MetS reported a two-fold increase in UCB risk (95 % CI: 1.38-3.19 - Table 2). Accordingly, in this study population, 8.1 % (95 % CI: 3.9-12.4 %) of all UCB cases were attributable to MetS (data not shown). Furthermore, 54 UCB cases (65.1 %) and 16 controls (38.1 %) with MetS reported diabetes; among these, the risk of UCB was 3.63 (95 % CI: 1.99-6.61) compared to those without MetS (Table 2). People with MetS without diabetes still showed a 16 % increased risk of UCB, but the association was not statistically significant.

The association between the MetS and UCB risk was similar in strata of gender (men vs. women; *P for heterogeneity = 0.08*), age (<65 years vs. ≥65 years; *P = 0.59*), education (<7 vs. ≥7 years; *P = 0.92*), geographical area (North vs. South of Italy; *P = 0.43*), and smoking habits (never, ever <20 cigarettes/day, and ever ≥20 cigarettes/day; *P = 0.51 -* Table 3). The association between the MetS and UCB risk was stronger (*P = 0.03*) for papillary UCB (OR = 2.61; 95 % CI: 1.68-4.04) than for non-papillary UCB (OR = 0.86; 95 % CI: 0.36-2.09), whereas no difference emerged according to tumour invasiveness (pTa/Tis vs. pT1-T4; *P = 0.69 -* Table 3). These results did not remarkably change using BMI ≥ 30 kg/m^2^ when the information of waist circumference was missing (data not shown).

**Table 3:** Odds ratio (OR) and corresponding 95 % confidence interval (CI) for urothelial carcinoma of the bladder (UCB), according to indicators of the metabolic syndrome (MetS) in strata of selected variables. Italy, 2003-2014

Variables	Indicator of MetS		OR (95 % CI)^a^	*χ*^2^ for heterogeneity; *p*-value
	No	Yes		
	Ca:Co	Ca:Co		
Sex				
Men	381:359	75:32	2.49 (1.56-3.96)	
Women	69:72	8:10	0.81 (0.25-2.60)	3.08; *P = 0.08*
Age (years)				
<65	183:199	25:14	2.53 (1.21-5.29)	
≥65	267:232	58:28	1.97 (1.18-3.29)	0.31; *P = 0.59*
Education (years)^b^				
<7	178:185	43:24	2.07 (1.14-3.75)	
≥7	271:246	40:18	2.16 (1.17-3.97)	0.01; *P = 0.92*
Geographical area				
North	307:311	64:31	2.31 (1.42-3.74)	
South	143:120	19:11	1.56 (0.66-3.67)	0.61; *P = 0.43*
Smoking habit^b^				
Never	67:150	7:16	1.16 (0.43-3.14)	
Ever <20 cig./day	190:174	34:13	2.45 (1.23-4.91)	
Ever ≥20 cig./day	186:104	41:13	1.85 (0.93-3.70)	1.37; *P = 0.51*
Histological subtype^b^				
Non-papillary	82:431	7:42	0.86 (0.36-2.09)	
Papillary	299:431	69:42	2.61 (1.68-4.04)	4.82; *P = 0.03*
Invasiveness^b^				
pTa/Tis	183:431	41:42	2.32 (1.42-3.79)	
pT1-T4	221:431	38:42	2.13 (1.28-3.57)	0.16; *P = 0.69*

^a^Adjusted for sex, age (<55; 55-59; 60-64; 65-69; 70-74; ≥75 years), study centre, education (<7; 7-11; ≥12 years), and tobacco smoking (never; former; current: <20; 20+ cigarettes/day); ^b^The sum does not add up to the total because of missing values

## Discussion

The present study supports a positive association between the MetS and risk of UCB, with a possible stronger association for papillary UCB. Conversely, no significant difference emerged according to gender, age, education, geographical area, and smoking habits. These findings are particularly interesting, giving the increasing prevalence of MetS worldwide and the attention by the scientific community on its effects on various health outcomes, including bladder and other urological cancers [6, 17].

In a prospective cohort study of 580 000 people – carried out within the Me-Can study – Haggstrom et al. [18] showed that MetS was associated with a significantly increased risk of UCB in men (RR = 1.10; 95 % CI: 1.01–1.18 for each incremental MetS unit), whereas no association was observed in women. Similarly, an Italian population-based study [19] observed a modest, non significant, increased risk of UCB only in men concurrently treated with antihyperglycaemic, antihypertensive, and hypolipidemic drugs. In their meta-analysis, Esposito et al. estimated that, in men, the presence of the MetS was significantly associated with the presence of UCB with a RR of 1.10 (95 % CI: 1.02–1.18) [11]. Our findings seem therefore to confirm this association with a substantially stronger OR as compared to previous studies [6, 7].

Mechanisms that link MetS and cancer risk are not fully understood. However, some MetS components have been extensively investigated as cancer risk factors. There is mounting evidence showing the negative influence of obesity on genitourinary malignancies [20]. Several epidemiological studies showed a positive relationship between obesity and an increased risk of UCB, although others did not find any significant associations [21, 22]. Although BMI is generally used to define the grade of obesity, in our study we used abdominal obesity since it better explains obesity-related health risk [23]. Moreover a recent study used visceral obesity as individual component of MetS to predict adverse pathological features in UCB [24].

The biological mechanism for obesity-related carcinogenesis is not yet well characterized, but many possibilities have been suggested. High levels of adipose tissue correlate with high levels of cholesterol, a precursor for the androgen testosterone, which stimulates epithelial cell proliferation. High adipose levels have also been correlated with high plasma levels of vascular endothelial growth factor (VEGF), which both stimulate proliferation of epithelial cells. Adipose tissue also secretes leptin, which has been implicated in enhancing angiogenesis and, consequently, may also enhance tumour development [25]. Adiposity has also been associated with reduced mitochondrial function and, in turn, increased circulating reactive oxygen species, which can cause DNA damage [26].

The strongest single risk factor found in the present study was diabetes mellitus. Nonetheless, people with the MetS but without diabetes had a 16 % increased risk of UCB. Furthermore, Table 2 shows a doubling of UCB risk in people with 4 MetS components (OR = 7.93; CI: 1.71-36.79) than in people with diabetes (OR = 3.63; CI: 1.99-6.61) suggesting that the MetS indeed plays a relevant role in the risk of UCB.

Previous cohort studies have investigated the association between diabetesand UCB risk [27–31]. All these studies reported an increased UCB risk for both men and women with diabeteslevels, but strongest associations were seen with longer diabetes duration [27,]. The Me-Can study reported a statistically significant increased risk among women, with an RR of 1.45 (95 % CI: 1.05–2.01) per mmol increment of glucose [32]. A possible additional pathway between diabetes and UCB risk is the increased incidence of urinary tract infections among subjects with diabetes [30]. Among separate components of MetS, high blood pressure and hypercholesterolemia were not significantly associated with the risk of UCB in our study. These findings are consistent with those of previous prospective studies [6, 18].

Possible study limitations included selection and information bias. The proportion of pTa/Tis in our case series (45.3 %) is slightly lower than expected (approximately 60 %), thus limiting the generalization of our results. However, similar associations were found for pTa/Tis and pT1-T4 UCB, suggesting that this type of selection bias had a limited impact on our results. Information on MetS components was based on self-reported data from a questionnaire, which collected history of diabetes, treated hypertension, and treated hyperlipidaemia, rather than direct measurements of fasting plasma glucose, blood pressure, triglycerides and HDL cholesterol. Underestimation of the prevalence of MetS may therefore have occurred. However, reliability of our questionnaire on diabetes was tested among almost 300 subjects who were interviewed twice, reporting a satisfactory agreement (k statistic = 0.85) [14]. Moreover, a recent cohort study from Spain showed that self-reported data on MetS indicators and on MetS itself are sufficiently accurate for epidemiological inference [32]. Likewise, validation studies of hypertension confirmed with a medical examination found a reasonable accuracy of self-reported information [33]. Other self-reported MetS indicators might have been underestimated, but such information bias is likely to have occurred similarly in cases and controls, and, consequently, should have led to an attenuation of the real association [16, 34].

Other potential limitations of this study design comprise recall bias, since cases might have been more sensitized than controls to report history of disease. The hospital setting should have, however, improved information comparability of cases to controls, because both groups were interviewed under similar conditions and were therefore similarly sensitized toward recalling medical history. However, cases and controls were enrolled from the same catchment areas, and careful attention was paid to exclude from the control group subjects admitted for any condition related to the exposures under study, including tobacco smoking. Furthermore, results were consistent when analyses were performed excluding, in turn, the main diagnostic categories of controls. On the other hand, our findings were strengthened by the nearly complete participation of identified cases and controls, and by the use of a validated, reproducible questionnaire [14].

## Conclusion

Our data suggest that metabolic aberrations related to the MetS, which are known to increase the risk of several types of cancer, also increase the risk of UCB. Both the worldwide increasing MetS prevalence and the rising incidence of UCB suggest that each year a considerable fraction of this cancer is attributable to the MetS. Thus, evidence is needed to investigate whether effective interventions to reduce the prevalence of the MetS in adult populations could reduce UCB risk. Moreover, patients with the MetS, even in presence of obesity and/or diabetes, should be encouraged to follow appropriate cancer control strategies.

## Abbreviations

BMI: Body mass index
Ca:Co: Cases:Controls
CI: Confidence interval
Cig: Cigarettes
HBV: Hepatitis b virus
HCV: Hepatitis c virus
IDF: International Diabetes Federation
MetS: Metabolic syndrome
OR: Odds ratio
PAR: Percent attributable risk
UCB: Urothelial carcinoma of the bladder
VEGF: Vascular endothelial growth factor
